# Supplementary material for: The IgA in milk induced by SARS-CoV-2 infection is comprised of mainly secretory antibody that is neutralizing and highly durable over time
Source: PLoS One. 2022 Mar 9;17(3):e0249723. doi: 10.1371/journal.pone.0249723 (PMC8906612; doi:10.1371/journal.pone.0249723)
Supplement: S1 Fig — (A) Secretory Ab versus IgG. (B) IgA versus IgG. Endpoint titers were used in 2-tailed Spearman correlation tests. SC: secretory component. (PDF) [file pone.0249723.s001.pdf]

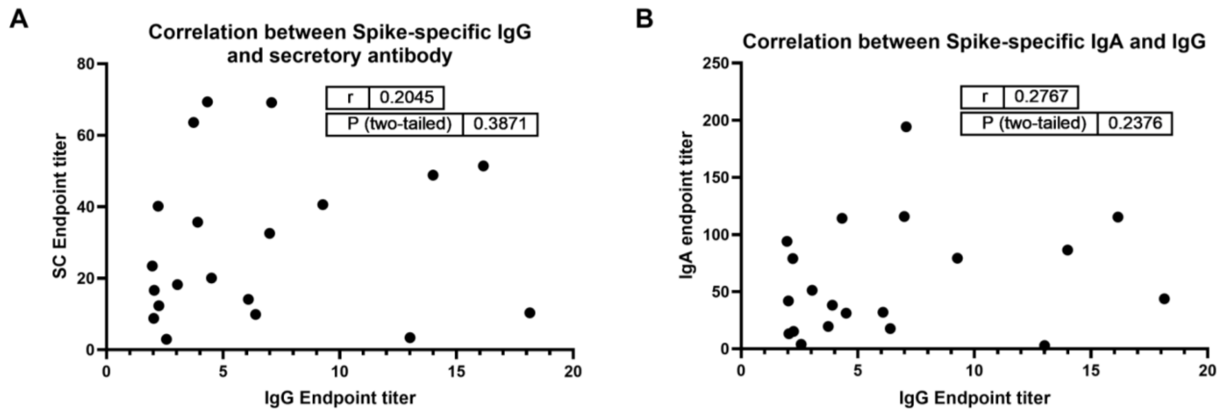


**S1 Fig 1. No correlation was found between Spike-specific milk IgG and IgA or IgG and secretory antibody titers.** (A) Secretory Ab versus IgG. (B) IgA versus IgG. Endpoint titers were used in 2-tailed Spearman correlation tests. SC: secretory component.
